# Trends in the Management of Small HER2-Positive Breast Cancers

**DOI:** 10.1245/s10434-025-17430-6

**Published:** 2025-05-13

**Authors:** Carolin Mueller, Rahul Rangan, Megan Kruse, Zahraa Al-Hilli

**Affiliations:** 1 Outcomes Research Consortium, Department of Anesthesiology, Cleveland Clinic, Cleveland, OH USA; 2 Department of Gynecology and Obstetrics, Comprehensive Cancer Center Erlangen-EMN (CCC ER-EMN), Universitätsklinikum Erlangen, Friedrich-Alexander-Universität Erlangen-Nürnberg (FAU), Erlangen, Germany; 3 Breast Center, Integrated Surgical Institute, Cleveland Clinic, Cleveland, OH USA; 4 Department of Hematology and Medical Oncology, Division of Breast Medical Oncology, Taussig Cancer Institute, Cleveland Clinic, Cleveland, OH USA

**Keywords:** Breast cancer, HER2 positive, Neoadjuvant systemic therapy, Adjuvant systemic therapy, Treatment patterns

## Abstract

**Background:**

The treatment approach for small HER2-positive (+) breast cancers seeks to optimize efficacy while minimizing potential overtreatment and associated toxicities. This study aims to evaluate recent trends in treatment patterns for small HER2+ tumors.

**Methods:**

Patients diagnosed with HER2+, cT1, cN0/pN0 breast cancer treated at a single institution from January 2018 to December 2022 were included. Clinicopathological, treatment, and follow-up data were collected and analyzed.

**Patients and Results:**

A total of 207 patients were included. Mean age was 63 (± 12.0) years. *T* category included cT1a in 12.1% (*n* = 25), cT1b in 28.0% (*n* = 58), and cT1c in 57.5% (*n* = 119), while 2.4% (*n* = 5) had clinical *T*1 category without further specification. Moreover, 74.4% (*n* = 154) were hormone receptor positive. Also, 66.7% (*n* = 138) received adjuvant therapy, 12.6% (*n* = 26) received neoadjuvant systemic therapy (NAT), and 12.1% (*n* = 25) received no systemic therapy. Administered regimens included: trastuzumab monotherapy in 6.1% (*n* = 10), taxane/trastuzumab in 55.5% (*n* = 91), taxane/carboplatin/trastuzumab in 18.9% (*n* = 31), and taxane/carboplatin/trastuzumab/pertuzumab in 15.2% (*n* = 25). In the 26 patients who received NAT, pathological complete response (pCR) was noted in 69.2% (*n* = 18). Overall, use of NAT increased from 2018 (7.1%) to 2021 (30.2%) and then decreased in 2022 (9.1%). The overall mastectomy rate was 35.3% (*n* = 73). Young age and multiple tumors were associated with a higher rate of mastectomy (age *p* < 0.001; multiple tumors *p* = 0.006). Upstaging of clinically node-negative patients occurred in 14.1% of patients at surgery.

**Conclusion:**

The treatment for cT1N0 HER2+ breast cancers includes primary surgery with adjuvant HER2-targeted therapy in combination with chemotherapy. Primary surgery may allow for an opportunity to deescalate adjuvant therapy with no impact on surgical plan.

**Supplementary Information:**

The online version contains supplementary material available at 10.1245/s10434-025-17430-6.

HER2-positive (+) breast cancers represent ~ 15% of all breast cancers.^1^ Early data suggested that women diagnosed with HER2+ early breast cancer faced an elevated risk of recurrence and experienced poorer survival outcomes compared with those with HER2-negative disease.^2^ For many years chemotherapy with anthracyclines and taxanes was standard of care for all patients with HER2+ breast cancer. However, during the last two decades, enhanced comprehension of tumor biology and the subsequent introduction of targeted therapies have significantly altered the approach to managing HER2+ breast cancer and led to improved survival rates.^3^

Trastuzumab revolutionized the therapy of HER2+ breast cancer.^4,5^ Its use in combination with adjuvant chemotherapy reduced the rates of recurrence by half among women with operable HER2+ breast cancer (hazard ratio (HR) 0.48; 95% confidence interval (CI) 0.39–0.59; *p* < 0.0001) and led to Food and Drug Administration (FDA) approval in 2006.^5^ In the TRYPHAENA and neoSPHERE studies, addition of pertuzumab in the neoadjuvant setting resulted in an even higher rate of tumor response, leading to FDA approval in 2013.^6–9^ Consequently, rates of neoadjuvant chemotherapy in HER2+, early breast cancer increased.^10^ In the adjuvant setting, the APHINITY trial showed improved invasive disease-free survival in patients with HER2+, operable breast cancer (node-positive or high-risk node-negative) when pertuzumab was added to standard adjuvant chemotherapy with trastuzumab.^7^ At a median follow-up of 8.4 years, the overall survival benefit of adjuvant pertuzumab has been established for node-positive patients.^11^

In recent years, new efforts to improve the treatment in HER2+ early breast cancer have been made. The KATHERINE trial assessed the administration of trastuzumab emtansine (T-DM1) in patients with residual disease after neoadjuvant chemotherapy.^12^ Risk of invasive disease recurrence was reduced by 50% in patients who received post-neoadjuvant therapy with T-DM1, making this a new treatment standard.^12^ The personalized approach in the post-neoadjuvant setting clearly favors neoadjuvant systemic therapy (NAT) for HER2+ early breast cancer. However, a major challenge is distinguishing between patients for whom standard therapy might be insufficient and those who may be overtreated and would benefit equally with less toxicity.^13^ This is especially relevant for small, node-negative tumors as survival outcomes for these patients have proven excellent with deescalated systemic therapy.^14–16^ For example, in the adjuvant setting, the APT study of weekly paclitaxel and trastuzumab followed by single-agent trastuzumab showed excellent 3-year disease-free survival rates (98.7%) and 10-year overall survival rates (94.3%) in stage I (*T* < 3 cm, N0) HER2+ tumors.^14,15^

This study sought to examine current treatment trends among HER2+ patients, with small tumors (T1a/b/c) and node-negative (N0) disease. We aimed to compare the utilization of systemic therapy (neoadjuvant versus adjuvant) on the basis of clinical tumor size and analyze potential factors influencing this decision. Moreover, surgical trends and outcomes were evaluated.

## Patients and Methods

### Study Population

This is a retrospective, institutional review board-approved, single-institution cohort study. Waiver of consent was approved by the institutional review board. All patients ≥ 18 years who were diagnosed with HER2+, clinical (c) tumor category T1(a–c), node-negative (N0) breast cancer treated from January 2018 to December 2022 were included. The 8th edition of the American Joint Committee of Cancer (AJCC) was used to determine the clinical tumor category.^17^ All patients received preoperative imaging including mammography, and ultrasound, as well as magnetic resonance imaging at the discretion of the treating physician. The largest single contiguous dimension of a tumor focus on preoperative imaging (mammography, ultrasound, or magnetic resonance imaging) was used to define the clinical tumor category. For multicentric tumors, the largest tumor was used to determine the clinical tumor size. Small satellite tumor foci of noncontiguous tumor were not included in the measurement of maximum tumor size.^17^

Only patients with clinically as well as pathologically node-negative disease were included, as patients with clinically or pathologically node-positive disease are likely to receive NAT or more “aggressive” systemic therapy regimens. Exclusion of this cohort addresses this bias; 41 patients with cN+ were excluded as well as 34 patients with pathologically positive nodes identified after primary surgery. Patients with isolated tumor cells (pN0(i+), malignant cell clusters no larger than 0.2 mm) were defined as N0 and therefore included in the analysis. Patients with prior history of breast cancer and those with distant metastases (M1) were excluded. HER2 positivity was determined by immunohistochemistry (IHC) on the initial diagnostic core biopsy specimen of 3+, or 2+ and fluorescence in situ hybridization (FISH) positive as per American Society of Clinical Oncology/College of American Pathologists (ASCO/CAP) guidelines. Pathologic complete response (pCR) was defined as no invasive disease in the breast and the axilla (ypT0/ypTis and ypTN0).^18^ Information on demographics, clinicopathological data, treatment data, and follow-up data was retrieved from the electronic medical record.

### Statistical Analysis

Analysis was performed using SPSS 28.0 (IBM, Armonk, USA). The Kolmogorov–Smirnov test was used to test for normal distribution in quantitative parameters. Consequently, quantitative parameters are presented as mean with standard deviation (if normally distributed) or median with minimum and maximum (if not normally distributed). Qualitative parameters are presented as absolute frequencies and percentages. Multiple logistic regression was performed to analyze treatment patterns in administration of neoadjuvant/adjuvant chemotherapy between the different years (2018–2022) and adjusted for clinical T category (cT1a/b versus cT1c), patient age, presence of multiple tumors, ethnicity, race, estrogen receptor (positive versus negative), and grading (G1/G2 versus G3). Additionally, a multiple logistic regression analysis was conducted to identify factors associated with the surgical treatment decision (mastectomy versus lumpectomy). Analyzed factors included year of treatment, clinical T category (cT1a/b versus cT1c), patient age, presence of multiple tumors, ethnicity, race, and administration of systemic therapy (neoadjuvant versus adjuvant). Results are reported as odds ratios and 95% confidence intervals. Number of excised lymph nodes in patients with neoadjuvant versus adjuvant therapy were compared using the Mann–Whitney *U* test.

## Results

**Fig. 1:**
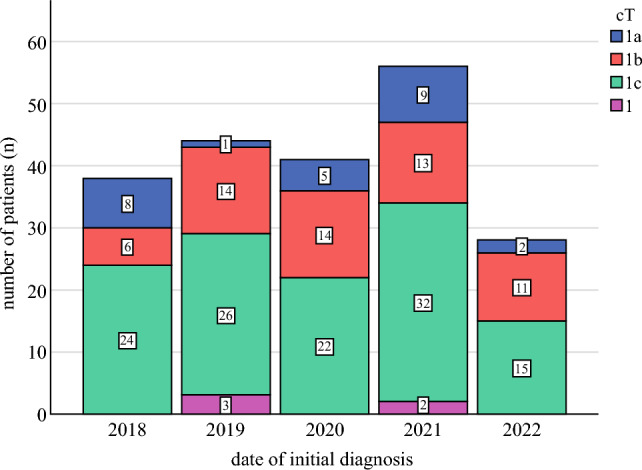
Clinical tumor category (cT) by year (2018–2022) in *n* = 207 patients (100%) with T1N0 HER2-positive breast cancer treated at the Cleveland Clinic between January 2018 and December 2022

A total of 207 patients (cT1N0) met inclusion criteria. Mean patient age was 63 (±12.0) years. Patient tumor and therapy characteristics are presented in Table 1. T category included cT1a in 12.1% (*n* = 25), cT1b in 28.0% (*n* = 58), and cT1c in 57.5% (*n* = 119). In 2.4% of patients (*n* = 5), clinical T1 category could not be specified as three patients only showed calcifications on mammography prior to therapy (no specific tumor size) and two patients had imaging at other hospitals that was not available for review. Clinical tumor category (cT) by year 2018–2022 is illustrated in Fig. 1. One hundred fifty-four patients (74.4%) were hormone receptor positive. Multiple tumors were identified in 18.8% of patients (*n* = 39): 11.6% (*n* = 24) in the same breast, and 7.2% (*n* = 15) with bilateral disease.

**Table 1:** Cohort description

Demographics		
	Number of patients (*n* = 207)	Percentage (%)
*Ethnicity*		
Hispanic or latinx	6	2.9%
Not hispanic or latinx	200	96.6%
Unknown	1	0.5%
*Race*		
White	179	86.5%
Black or African American	21	10.1%
Native Hawaiian or Other Pacific Islander	1	0.5%
Other	4	1.9%
Unknown	2	1.0%
**Tumor characteristics**		
*Clinical tumor category (cT)*		
cT1a	25	12.1%
cT1b	58	28.0%
cT1c	119	57.5%
cT1*	5	2.4%
*Clinical tumor category(cN)*		
cN0	207	100%
*Grade*		
G1	9	4.4%
G2	88	42.5%
G3	104	50.2%
GX (unknown)	6	2.9%
*Biomarkers*		
ER positive	154	74.4%
PR positive	114	55.1%
Multiple tumors**		
Same breast (multifocal)	24	11.6%
Both breasts (bilateral)	15	7.2%
*Histology*		
NST/ductal	183	88.4%
Lobular carcinoma	9	4.3%
Other***	15	7.3%
**Treatment characteristics**		
*Surgical procedure for breast*		
Mastectomy (with or without reconstruction)	73	35.3%
Lumpectomy	134	64.7%
*Radiation*		
Yes	119	57.5%
No	66	31.9%
Unknown	22	10.6%
*Endocrine therapy*		
Yes	141	68.1%
No	48	23.2%
Unknown	18	8.7%
*Endocrine therapy agent*		
Aromatase inhibitor	115	55.6%
Tamoxifen	22	10.6%
Exemestane	3	1.4%
Other	1	0.5%
*Systemic therapy summary*		
Neoadjuvant	26	12.6%
Adjuvant	138	66.7%
Total (neoadjuvant or adjuvant)	164	79.2%
No systemic therapy	25	12.1%
Unknown	18	8.7%

*G* grade, *ER* estrogen receptor, *PR* progesterone receptor, *NST* no special type

Numbers presented as frequencies (*n*) and percentages (%). All percentages refer to *n* = 207 patients (100%) with T1N0 HER2-positive breast cancer treated at the Cleveland Clinic between January 2018 and December 2022

*In five patients, clinical T1 category could not be specified. In three patients, only calcifications were detected prior therapy (no specific tumor size), and two patients received imaging at other hospitals

**Biggest tumor was < 2 cm (max. cT1c)

***Other included invasive micropapillary carcinoma, and mixed carcinomas

### Systemic Therapy

A total of 12.6% (*n* = 26) received NAT, 66.7% (*n* = 138) received adjuvant systemic therapy, and 12.1% (*n* = 25) did not receive any systemic therapy. Of the 25 patients who did not receive any systemic therapy, 64.0% (*n* = 16) chose not to proceed with systemic treatment after discussing and weighing the potential benefits and risks, 32.0% (*n* = 8) refused recommended treatment, and in 4.0% (*n* = 1) reasons for declining treatment are unknown.

**Fig. 2:**
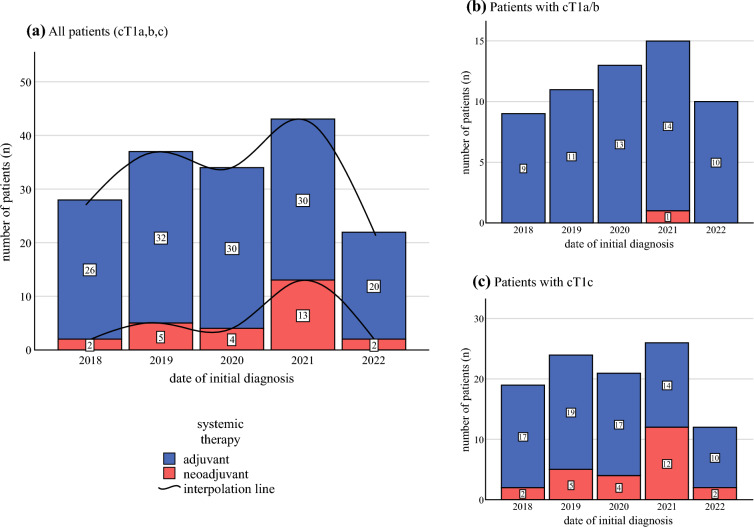
Administration of neoadjuvant and adjuvant therapy from 2018 to 2022 in all patients who received systemic treatment (*n* = 164) (**a**) and categorized into those with clinical T1a/b (*n* = 58) (**b**) and clinical T1c (*n* = 102) (**c**). In four patients who underwent systemic treatment, the specific clinical T1 category could not be determined

Systemic therapy included trastuzumab monotherapy in 6.1% (*n* = 10), taxane/trastuzumab (TH) in 55.5% (*n* = 91), taxane/carboplatin/trastuzumab (TCH) in 18.9% (*n* = 31), taxane/carboplatin/trastuzumab/pertuzumab (TCHP) in 15.2% (*n* = 25), and other regimens in 1.8% (*n* = 3). In the adjuvant setting, TH was most frequently administered (65.9%), whereas in the neoadjuvant setting most patients received TCHP (80.8%) (Table 2). NAT use increased from 2018 (7.1%) to 2021 (30.2%) then decreased in 2022 (9.1%) (Fig. 2a). Similar patterns were observed in patients with T1a/b (2021: 6.7%; 2022: 0%) and T1c tumors (2021: 46.2%; 2022: 16.7%) (Fig. 2b, c).

**Table 2:** Summary of systemic therapy received by cohort

Systemic therapy	All patients (*n* = 164)	Neoadjuvant (*n* = 26)	Adjuvant (*n* = 138)
*Systemic therapy agents*			
Trastuzumab monotherapy	10 (6.1%)	0 (0.0%)	10 (7.2%)
Taxane + trastuzumab	91 (55.5%)	0 (0.0%)	91 (65.9%)
Taxane + carboplatin + trastuzumab	31 (18.9%)	3 (11.6%)	28 (20.3%)
Taxane + carboplatin + trastuzumab + pertuzumab	25 (15.2%)	21 (80.8%)	4 (2.9%)
Other	3 (1.8%)	1 (3.8%)	2 (1.4%)
Unknown	4 (2.4%)	1 (3.8%)	3 (2.2%)
*Early discontinuation of systemic therapy*			
Discontinuation of chemotherapy prior to planned completion	32 (19.5%)	7 (26.9%)	25 (18.1%)
Discontinued systemic therapy agent**			
Taxol	21 (65.6%)	5 (71.4%)	16 (64.0%)
Platinum	4 (12.5%)	2 (28.6%)	2 (8.0%)
Trastuzumab	7 (21.9%)	0 (0.0 %)	7 (28.0)
Pertuzumab	3 (9.4%)	1 (14.3%)	2 (8.0%)
Other	2 (6.3%)	1 (14.3%)	1 (4.0%)

Numbers given as frequencies (*n*) and percentages (%)

**Some patients discontinued more than one agent (in the neoadjuvant setting two patients, in the adjuvant setting three patients). Percentages refer to the patients in each category who discontinued early (*n* = 32 for all patients, *n* = 7 for neoadjuvant, and *n* = 25 for adjuvant)

Overall, discontinuation of systemic therapy occurred in 19.5% (*n* = 32/164) of patients and dose reduction was observed in 21.5% (*n* = 32/149). Patients with NAT discontinued more often compared with patients who received the systemic therapy in the adjuvant setting (26.9% versus 18.1%, respectively) (Table 2).

Of the patients treated with NAT, pCR was noted in 69.2% (*n* = 18/26). Of these, ten patients were hormone receptor positive (10/17: 58.8%) and eight patients (8/9: 88.9%) were hormone receptor negative.

Multiple logistic regression was performed to analyze treatment patterns in administration of neoadjuvant versus adjuvant systemic therapy between the different years (2018–2022) in T1a/b/cN0 patients. The year of treatment was associated with treatment choice (*p* = 0.016), where patients received NAT more frequently in 2021. Clinical tumor category cT, age, and presence of multiple tumors had significant influence on treatment timing (*p* = 0.004, *p* = 0.002, and *p* = 0.016, respectively), whereas race, ethnicity, estrogen receptor, and grading were not associated with timing of treatment (*p* = 0.894, *p* = 0.074, *p* = 0.240, and *p* = 0.169, respectively) (Table 3).

**Table 3:** Systemic therapy treatment patterns by year of treatment and patient demographics

	Odds ratio	95% confidence interval	*p* Value
*Year of treatment* 2018 (reference) 2019 2020 2021 2022	1 2.95 4.27 20.36 2.54	— (0.35; 24.70) (0.49; 37.37) (2.68; 154.84) (0.22; 29.54)	Wald test: **0.016** 0.319 0.190 **0.004** 0.456
cT (cT1ab vs. cT1c)	47.71	(3.50; 650.82)	**0.004**
Age	0.92	(0.87; 0.97)	**0.002**
Multiple tumors (same or contralateral breast)	4.31	(1.31; 14.19)	**0.016**
Race	1.14	(0.18; 7.30)	0.894
Ethnicity	13.93	(0.77; 250.93)	0.074
Estrogen receptor (positive versus negative)	0.45	(0.12; 1.71)	0.240
Grading (G1/G2 vs. G3)	0.43	(0.13; 1.43)	0.17

Multiple logistic regression was performed to analyze treatment patterns in administration of neoadjuvant/adjuvant systemic therapy between the different years (2018–2022). The model was adjusted for clinical T category cT (cT1ab versus cT1c), patient age, race (white versus other), ethnicity (Hispanic/Latinx versus no Hispanic/Latinx), multiple tumors (yes/no), estrogen receptor (positive versus negative), and grading (G1/G2 versus G3)

### Breast and Axillary Surgery

Breast surgery type included lumpectomy in 64.7% (*n* = 134) and mastectomy with or without reconstruction in 35.3% (*n* = 73). The lumpectomy rate per year remained stable over time (Table 4). Multiple logistic regression was performed to analyze factors influencing the surgical treatment decision (mastectomy versus breast-conserving surgery). Younger patients as well as patients with multiple tumors were more likely to have a mastectomy (age: *p* < 0.001; multiple tumors: *p* = 0.006, respectively). Year of treatment, clinical tumor category cT (cT1a/b versus cT1c), and race or ethnicity had no significant influence on surgery type. Moreover, timing of systemic therapy (neoadjuvant versus adjuvant) was not associated with breast surgery type (Table 5).

**Table 4:** Surgical trends by year of diagnosis

	2018	2019	2020	2021	2022
*All patients*					
Mastectomy	16 (42.1%)	12 (27.3%)	18 (43.9%)	19 (33.9%)	8 (28.6%)
Lumpectomy	22 (57.9%)	32 (72.7%)	23 (56.1%)	37 (66.1%)	20 (71.4%)
*Neoadjuvant*					
Mastectomy	1 (50.0%)	1 (20.0%)	2 (50.0%)	6 (46.2%)	2 (100%)
Lumpectomy	1 (50.0%)	4 (80.0%)	2 (50.0%)	7 (53.8%)	0 (0%)
*Adjuvant*					
Mastectomy	9 (34.6%)	10 (31.3%)	13 (43.3%)	8 (26.7%)	5 (25.0%)
Lumpectomy	17 (65.4%)	22 (68.7%)	17 (56.7%)	22 (73.3%)	15 (75.0%)

**Table 5:** Surgical treatment patterns by year of treatment and patient demographics

	Odds ratio	95% confidence interval	*P *Value
*Year of treatment* 2018 (reference) 2019 2020 2021 2022	1 0.585 1.861 0.982 0.773	– (0.17; 1.98) (0.59; 5.86) (0.31; 3.16) (0.20; 2.97)	Wald test: 0.37 0.389 0.288 0.975 0.708
cT (cT1ab vs. cT1c)	1.24	(0.55; 2.82)	0.606
Age	0.939	(0.91; 0.97)	**< 0.001**
Multiple tumors (same or contralateral breast)	3.669	(1.46; 9.24)	**0.006**
Race	0.515	(0.15; 1.82)	0.302
Ethnicity	2.046	(0.21; 20.14)	0.540
Systemic therapy (neoadjuvant vs. adjuvant)	0.727	(0.24; 2.20)	0.572

Multiple logistic regression was performed to analyze rates of mastectomy versus lumpectomy between the different years (2018–2022). The model was adjusted for clinical T category (cT1ab versus cT1c), patient age, race (white versus other), ethnicity (Hispanic/Latinx versus no Hispanic/Latinx), multiple tumors (yes/no), and administration of systemic therapy (neoadjuvant versus adjuvant)

All patients had sentinel node biopsy. Of all cN0 patients identified initially for inclusion (*n* = 241) and undergoing primary surgery, *n* = 34 patients were found to have node-positive disease at surgery (14.1%) and were excluded from the prior analysis as stated in the “Patients and Methods” section. Tumor as well as treatment characteristics for this cohort are presented in Supplementary Table 1. For these patients, the majority (85.3%, 29/34) avoided completion axillary lymph node dissection due to satisfying criteria for omission further axillary surgery.^19–21^ Of the patients undergoing NAT, no patients were found to have ypN+ disease. A total of 2.6 (±1.7) axillary lymph nodes were removed per patient. More lymph nodes were removed in patients undergoing NAT compared with the adjuvant treatment group (3.8 (±2.6) versus 2.4 (±1.4), *p* = 0.004).

## Discussion

Our study shows that the use of NAT for cT1N0 HER2+ breast cancer increased from 2018 until 2021 but declined again in 2022. This trend can be explained by change in practice patterns related to high-impact publications from clinical trials assessing optimization and “right-sized” approach for HER2+ tumors given excellent outcomes with contemporary systemic therapy. Furthermore, our study demonstrates that primary surgery in patients with cT1cN0 HER2+ disease may allow for deescalation in systemic therapy in the majority of cases (85.9%) with no impact on breast-conserving surgery or axillary lymph node dissection rates.

After the NeoSphere and TRYPHAENA study were published in 2012 and 2013, showing that dual antibody blockade with trastuzumab and pertuzumab in the neoadjuvant setting led to improved disease-free survival in early-stage HER2+ cancers, the rate of NAT increased.^8–10^ The response to NAT gives valuable prognostic information, as pCR is associated with better recurrence-free and overall survival.^22^ This allows clinicians to optimize adjuvant treatment in accordance with risk category. Furthermore, one may expect to see an increase in utilization of NAT for HER2+ tumors after publication of the KATHERINE trial in the beginning of 2019.^12^ The trial led to the approval of T-DM1 in the adjuvant setting for patients who did not achieve pCR after NAT.^12^ In the present study, this trend toward NAT peaked in 2021, when a total of 30.2% of clinical T1N0 patients received NAT. However, the KATHERINE study enrolled patients with cT1–T4, nodal category N0–N3, and no metastasis, but cT1aN0 and T1bN0 were excluded.^12^ Therefore, NAT was not recommended for patients with T1aN0 and T1bN0 by the ASCO expert panel in 2021.^23^ In our analysis, only one patient with cT1a/bN0 received NAT in 2021.

Other studies that might have contributed to the change in treatment patterns and deescalation for small HER2+ tumors are the APT and the WSG-ADAPT-HER2+/HR− trial.^14–16^ In the APT study, patients received weekly adjuvant paclitaxel and trastuzumab followed by single-agent trastuzumab.^14,15^ Excellent 3-year disease-free survival rates (98.7%) and 10-year overall survival rates (94.3%) in stage I (*T* < 3 cm, N0) HER2+ tumors were demonstrated.^14,15^ Moreover, the WSG-ADAPT-HER2+/HR− trial (published in 2022) compared trastuzumab and pertuzumab with and without paclitaxel for 12 weeks in the neoadjuvant setting.^16^ For patients achieving pCR, the exclusion of further chemotherapy did not impact invasive disease-free survival.^16^ However, the study was limited to patients with hormone receptor-negative disease, as pCR rates are known to be higher for this cohort.^16^ Furthermore, the ATEMPT trial, published in 2021, investigated adjuvant T-DM1 versus paclitaxel and trastuzumab and showed excellent 3-year disease-free survival with T-DM1 only (97.8%).^24^ A chemotherapy-free approach in patients with HER2+, ER+ breast cancer was investigated in the NA-PHER2 study, an exploratory phase 2 trial.^25,26^ Patients with clinical tumor category cT1c–T4/N0–2 received NAT with trastuzumab and pertuzumab plus palbociclib and fulvestrant, which led to decreased mean Ki67 levels, and a clinical objective response was seen in 97% of patients (*n* = 29).^25,26^ Furthermore, therapy response to antibody treatment (trastuzumab and pertuzumab) in patients with early breast cancer (stage I–IIIA) using ^18^F-fluorodeoxyglucose (FDG) positron emission tomography (PET) was studied in the PHERGain trial, a multicentric randomized phase II trial.^27,28^ On the basis of ^18^F-FDG PET results, patients either continued dual HER2 blockade if they responded to treatment or switched to chemotherapy if they were nonresponders.^27,28^ The 3-year invasive DFS was excellent when response to antibody treatment was noted via ^18^F-FDG PET and therefore identified a cohort for whom it might be safe to omit chemotherapy.^28^

The results of trials to date prompted increasing consideration of whether “all patients” genuinely experience benefits from the therapy or if employing less treatment could yield comparable effectiveness while minimizing toxicity.^13,29^ A meta-analysis of eight randomized controlled trials of HER2+ malignancies (seven breast cancer and one gastroesophageal) including a total of 8420 patients indicated that addition of pertuzumab to chemotherapy plus trastuzumab increased the risk of clinical heart failure (risk ratio (RR) [95% CI]: 1.97 [1.05–3.70]; *I*^2^ = 0%).^31^ However, as the absolute risk for heart failure was low (8/1000 patients), the authors did not recommend omitting pertuzumab in eligible patients.^30^ Furthermore, an elevated risk for diarrhea (RR 2.26, 95% CI 1.87–2.74) in patients receiving dual blockade of pertuzumab plus trastuzumab is described.^31^ Regarding carboplatin, another meta-analysis of 11,049 patients focused on NAT in HER2+ early breast cancer. The study showed that addition of carboplatin to pertuzumab/trastuzumab/docetaxel led to a higher risk of thrombocytopenia grade ≥ 3 (12.9% with TCHP versus 0% with THP).^32^. However, irrespective of hormone receptor expression, TCHP was associated with a higher rate of pCR compared with THP (OR 1.45, 95% CI 0.73–2.88).^32^ Taken together, these data support a balance that maximizes effectiveness while reducing toxicity. Consequently, current and ongoing trials are exploring deescalation strategies in minimizing the use of chemotherapy in early-stage breast cancer (e.g., ATEMPT 2.0 trial (NCT04893109)). Our study showed a higher rate of treatment discontinuation in the neoadjuvant setting compared with those receiving a deescalated regimen in the adjuvant setting. On the other hand, another challenge is identifying patients for whom deescalated therapy may be insufficient. A cohort study of the National Cancer Database involving approximately 7000 T1–2/N0 HER2+ patients revealed that those without pCR after neoadjuvant chemotherapy had poor outcomes.^33^ This finding highlights that the neoadjuvant approach might be important to identify nonresponders, who could potentially benefit from intensified treatment.^33^ At present, there is no mechanism by which to stratify T1N0 tumors that aids in identification of those best suited to receive neoadjuvant therapy as a means to optimize adjuvant therapy. In the future, on the basis of the results of the ATEMPT and ATEMPT 2.0 trials, we may see more utilization of T-DM1 as the backbone of management of T1N0 tumors, which would eliminate the need to give neoadjuvant systemic therapy for patients to receive this agent.

However, the general benefit of extensive systemic treatment for small tumors (< 1 cm) is questionable, as the risk for distant and local recurrence is low. A retrospective analysis showed that the 5-year distant-recurrence-free survival for pT1b tumors was 97%, and for pT1a 99%.^34^ However, another analysis from Finland showed that distant-disease-free survival after 10 years was 84% in patients with HER2+ pT1a/b N0 tumors who did not receive systemic anti-HER2 therapy.^35^ Even though the benefit of trastuzumab in cT1a/b tumors has not been studied in randomized phase III clinical trials, retrospective studies suggest better disease-free and overall survival after adjuvant trastuzumab treatment (at least in cT1b tumors).^36–38^ An analysis of the National Clinical Database in Japan compared 5-year follow-up data in 2736 pT1N0/1M0 HER2+ patients, of whom 1472 were treated with systemic therapy (chemotherapy +/− trastuzumab) and 1265 received no systemic therapy.^37^ Systemic treatment led to enhanced overall survival in patients with pT1b/c breast cancer but not in those with pT1a node-negative HER2+ breast cancer.^37^ In the present study, 12.1% (*n* = 25) did not receive systemic therapy. Of these patients, 64.0% (16/25) had pT1a/pT1bN0 tumors, who chose not to proceed with systemic treatment after being informed and considering the pros and cons.

Regarding therapy regimens, administered systemic therapy agents used in our cohort are in line with current recommendations. When NAT is offered, combination of chemotherapy with trastuzumab +/− pertuzumab is recommended.^23^ In our study, 80.8% of patients received TCHP in the NAT setting, and 11.6% received TCH. In contrast, the most administered regimen in the adjuvant setting was TH (65.9%), followed by TCH (20.3%) (Table 2). A small minority (7.2%) received trastuzumab monotherapy or TCHP adjuvant (2.9%). This is also in line with current recommendations of the ASCO guidelines, where adjuvant trastuzumab in combination with (non-anthracycline) chemotherapy is recommended in patients with early-stage node-positive or node-negative (cT > 1 cm) breast cancer.^39^ It is worth noting that patients who underwent surgery first received less aggressive systemic therapy, making them less likely to discontinue their treatment (26.9% versus 18.1%, respectively).

With regard to surgical treatment, younger patients, as well as patients with multiple tumors were more likely to have a mastectomy in the present study. This is not surprising as, in young women, mastectomy is often regarded as the preferred intervention (regardless of tumor size), owing to concerns about the poorer prognosis associated with the disease in young patients.^40^ Moreover, patients’ preferences significantly influence the decision-making process, with the choice of mastectomy often driven by anxiety.^40^ Furthermore, the administration of NAT had no influence on mastectomy rates in the present study. This is in contrast to previous studies showing that NAT is an effective approach for reducing tumor size in early breast cancer, thereby enhancing the likelihood of breast-conserving surgery.^41^ The reason for this might be that our cohort included only patients with T1 tumors, which are inherently small and typically suitable for treatment with breast-conserving surgery. Moreover, we saw no change in trends of surgical treatment between the different treatment years (2018–2022). The rise in mastectomy rates within the neoadjuvant group in 2022 should be interpreted cautiously, as the group included only two patients. The 100% mastectomy rate is therefore most likely coincidental. Interestingly, we also observed that more lymph nodes were removed in patients who underwent NAT compared with those who received surgery first. This observation might be coincidental, as only patients with clinically negative lymph nodes were included.

Limitations of this study are mainly due to its retrospective nature, as it pertains to treatment details and deescalation. The short duration of follow-up hinders our ability to evaluate long-term oncologic outcomes, such as disease-free survival, rate of recurrence, or overall survival. Moreover, the coronavirus disease 2019 (COVID-19) pandemic may also have influenced changes in treatment patterns and recommendations by professional societies.^42,43^ In June 2020, for example, the COVID-19 Pandemic Breast Cancer Consortium published a recommendation emphasizing the need to minimize surgical resources by selectively postponing operations and prioritizing patients for alternative (systemic) therapies whenever feasible.^44^ As surgical procedures were postponed in many institutions, the use of neoadjuvant treatment, especially bridging with endocrine therapy where appropriate, increased.^42,45^ Furthermore, studies performed during that time demonstrated that fewer patients were diagnosed with early-stage disease as screening service programs paused.^42,45^ A single-center retrospective study from the USA saw also an increase in NAT for patients with stage I and II disease (12.4% in 2018 versus 20.7% in 2020).^46^ In contrast, data from the Netherlands Cancer registry of around 34,000 women saw a decline in NAT for stage I breast cancer in 2020 compared withb 2018/2019 (OR 0.24, 95% CI 0.11–0.53).^43^

In conclusion, a careful balance between the toxicity of treatment and its oncological benefits holds significant importance when deciding on neoadjuvant or adjuvant treatment recommendations for early-stage and small HER2+ breast cancer. Current recommendations state that node-positive or high-risk node-negative patients should be offered NAT,^23^ whereas stage I disease should be treated with primary surgery.^39^ Our study indicates that upstaging of clinically node-negative patients who underwent primary surgery is uncommon and occurs in about 14.1% of patients, suggesting that only a small number of patients would have significantly benefited from NAT over primary surgery. Additionally, having surgery first may allow for deescalation in adjuvant systemic treatment and an increase in the likelihood of completion of treatment. Therefore, primary surgery followed by adjuvant systemic therapy might be the preferred approach for cT1N0 HER2+ breast cancer.

## Supplementary Information

Below is the link to the electronic supplementary material.

Supplementary file1 (DOCX 18 kb)

## Data Availability

The datasets generated during the current study are available from the corresponding author on reasonable request.
