# Supplementary material for: Trends in the Management of Small HER2-Positive Breast Cancers
Source: Ann Surg Oncol. 2025 May 13;32(8):5510–20. doi: 10.1245/s10434-025-17430-6 (PMC12222400; doi:10.1245/s10434-025-17430-6)
Supplement: Supplementary file 1 — Supplementary file1 (DOCX 18 kb) [file 10434_2025_17430_MOESM1_ESM.docx]

**Supplementary Table 1:** Cohort description of patients with clinical T1 category and clinically node negative disease (cN0) who were found to be pathologically node positive (pN+).

| **Tumor characteristics** | | |
| --- | --- | --- |
| **Clinical Tumor Category cT** | **Number of patients (n=34)** | **Percentage (%)** |
| cT1a | 0 | 0 |
| cT1b | 8 | 23.5 |
| cT1c | 26 | 76.5 |
| **Pathological Tumor Category pN** | | |
| pN1mi* | 4 | 11.8 |
| pN1a | 29 | 85.3 |
| pN1b | 1 | 2.9 |
| **Grading** | | |
| G1 | 1 | 2.9 |
| G2 | 10 | 29.4 |
| G3 | 23 | 67.6 |
| **Receptors** | | |
| ER positive | 25 | 73.5 |
| PR positive | 21 | 61.8 |
| **Multiple Tumors**** | | |
| Same breast (multifocal) | 8 | 23.5 |
| Both breasts (bilateral) | 0 | 0 |
| **Histology** | | |
| NST / ductal | 31 | 91.2 |
| Lobular carcinoma | 3 | 8.8 |
| **Treatment characteristics** | | |
| **Surgical Procedure Breast** | | |
| Mastectomy  (With or without reconstruction) | 17 | 50.0 |
| Lumpectomy | 17 | 50.0 |
| **Surgical Procedure Axilla** | | |
| Sentinel-Node-Biopsy | 20 | 58.8 |
| Axillary Lymph Node Dissection  (With or without sentinel-node-biopsy) | 14 | 41.2 |
| **Radiation** | | |
| Yes | 27 | 79.4 |
| No | 7 | 20.6 |
| **Endocrine Therapy** | | |
| Yes | 23 | 67.6 |
| No | 10 | 29.4 |
| Unknown | 1 | 2.9 |
| **Endocrine Therapy Agent** | | |
| Aromatase inhibitor | 17 | 73.9 |
| Tamoxifen | 6 | 26.1 |
| Exemestane | 0 | 0 |
| **Systemic Therapy Summary** | | |
| Neoadjuvant | 0 | 0 |
| Adjuvant | 33 | 97.1 |
| No systemic therapy | 0 | 0 |
| Unknown | 1 | 2.9 |
| **Systemic Therapy Agent** | | |
| Trastuzumab | 0 | 0 |
| Taxane + trastuzumab | 3 | 8.8 |
| Trastuzumab + Pertuzumab | 1 | 2.9 |
| Taxane + carboplatin + trastuzumab | 7 | 20.6 |
| Taxane + carboplatin + trastuzumab + pertuzumab | 17 | 50.0 |
| Other | 1 | 2.9 |
| Unknown*** | 5 | 14.7 |

G: Grading, ER: estrogen receptor, PR: progesterone receptor, NST: no special type

Numbers are presented in frequencies (n) and percentages (%). All percentages refer to n=34 patients (100%) treated with T1 category and clinically node negative disease (cN0) who were found to be pathologically node positive (pN+) HER2 positive breast cancer at the Cleveland Clinic between January 2018 and December 2022.

*pN1mi: Micrometastases (approximately 200 cells, larger than 0.2 mm, but none larger than 2.0 mm)

**biggest tumor was < 2cm (max. cT1c)

***patients were treated at other institutions
